# Draft Genome Sequence of Ristocetin-Producing Strain *Amycolatopsis* sp. Strain MJM2582 Isolated in South Korea

**DOI:** 10.1128/genomeA.01091-14

**Published:** 2014-10-30

**Authors:** Min Jung Kwun, Jinhua Cheng, Seung Hwan Yang, Dong-Ryung Lee, Joo-Won Suh, Hee-Jeon Hong

**Affiliations:** a Department of Biochemistry, University of Cambridge, Cambridge, United Kingdom; b Center for Nutraceutical and Pharmaceutical Materials, Myongji University, Yongin, Republic of Korea; c Division of Bioscience and Bioinformatics, College of Natural Science, Myongji University, Yongin, Republic of Korea

## Abstract

The draft genome sequence of a ristocetin-producing *Amycolatopsis* strain (sp. MJM2582) isolated in South Korea is reported here. This strain has a genome of approximately 8.9 Mb containing 7,933 predicted genes, including the ristocetin cluster and 32 additional predicted secondary metabolite biosynthesis clusters.

## GENOME ANNOUNCEMENT

*Amycolatopsis* sp. MJM2582 was isolated from a rhizosphere sample from Ami Mountain in Chungcheongnam-do province (located in the western part of South Korea) during a screening for producers of glycopeptide antibiotics and was subsequently observed to produce ristocetin (1). Glycopeptides are an important class of antibiotics that are particularly effective against Gram-positive bacteria. This family also includes vancomycin, an antibiotic of last resort which is reserved for the treatment of serious infections that are resistant to other antibiotics, for example, methicillin-resistant *Staphylococcus aureus* (MRSA) (2). The sp. MJM2582 has been shown to produce a number of different ristocetin derivatives, but ristocetin A is the most abundant product (1). Ristocetin A is a type III glycopeptide antibiotic and possesses a distinctive branched tetrasaccharide structure. Strain *Amycolatopsis* sp. MJM2582 is therefore an alternative natural source of ristocetin to the original *Amycolatopsis lurida* isolate, which is the current commercial producer of ristocetin A (3). Although a toxic side effect on platelet aggregation led to the discontinuation of ristocetin A as a antimicrobial (4), the aglycone of ristocetin A has been found to be free of this undesirable activity and, more significantly, exhibits improved antimicrobial activity compared with the parent compound (5). Here, we report the draft sequence of *Amycolatopsis* sp. MJM2582, in which we have identified 33 secondary metabolite biosynthetic clusters, including the ristocetin A cluster (GenBank accession number KF882511) (1).

The genome of *Amycolatopsis* sp. MJM2582 was sequenced using Illumina MiSeq to a coverage depth of 83-fold and then assembled using the GS De Novo Assembler (Newbler software version 2.8). The assembled genome comprised a total of 13 large contigs. The average size of these large contigs is 687,568 bp, and the largest contig is 2,111,476 bp in length. The estimated size of the genome is 8,932,977 bp with an average G+C content of 68.84%. The draft genome sequence was annotated using the Prokaryotic Genome Annotation Pipeline (PGAP) with GeneMarkS+ (6, 7), which predicted 7,933 coding sequence (CDS) regions, 41 pseudogenes, 54 tRNAs, 14 noncoding RNAs (ncRNAs), and a single copy each of 5S rRNA, 23S rRNA, and 16S rRNA. The 16S rRNA sequence of *Amycolatopsis* sp. MJM2582 showed 99% identity to that of *A. lurida*, the producer of commercial ristocetin, but phylogenic analysis indicated that *A.* sp. CMU-PLA03 is actually taxonomically the most closely related strain to *Amycolatopsis* sp. MJM2582. AntiSMASH (8) was used to predict potential secondary metabolite synthetic gene clusters. In addition to the 39 open-reading frames (ORFs) of the ristocetin A cluster, gene clusters predicted to encode the production of lantipeptides, NRPS (nonribosomal peptide synthease) compounds, terpenes, type I polyketide antibiotics, bacteriocins, ectoine, and several unknown metabolites were identified.

### Nucleotide sequence accession numbers.

The draft genome sequence of *Amycolatopsis* sp. MJM2582 has been deposited in the DDBJ/EMBL/GenBank database under the accession number JPLW00000000. The version described in this paper is version JPLW01000000.
